# P-tau217 and other blood biomarkers of dementia: variation with time of day

**DOI:** 10.1038/s41398-024-03084-7

**Published:** 2024-09-13

**Authors:** Ciro della Monica, Victoria Revell, Giuseppe Atzori, Rhiannon Laban, Simon S. Skene, Amanda Heslegrave, Hana Hassanin, Ramin Nilforooshan, Henrik Zetterberg, Derk-Jan Dijk

**Affiliations:** 1 Surrey Sleep Research Centre, University of Surrey, Surrey, UK; 2 UK Dementia Research Institute Care Research & Technology Centre, Imperial College London and University of Surrey, Surrey, UK; 3 UK Dementia Research Institute at UCL, London, UK; 4 Surrey Clinical Trials Unit, University of Surrey, Surrey, UK; 5 Department of Neurodegenerative Disease, UCL Institute of Neurology, London, UK; 6 Surrey Clinical Research Facility, University of Surrey, Surrey, UK; 7 NIHR Royal Surrey CRF, Guildford, UK; 8 Surrey and Borders Partnership NHS Foundation Trust Surrey, Surrey, UK; 9 Department of Psychiatry and Neurochemistry, Institute of Neuroscience and Physiology, The Sahlgrenska Academy, University of Gothenburg, Mölndal, Sweden; 10 Clinical Neurochemistry Laboratory, Sahlgrenska University Hospital, Mölndal, Sweden; 11 Hong Kong Center for Neurodegenerative Diseases, Hong Kong, China; 12 Wisconsin Alzheimer’s Disease Research Center, University of Wisconsin School of Medicine and Public Health, University of Wisconsin-Madison, Madison, WI USA

**Keywords:** Physiology, Biomarkers

## Abstract

Plasma biomarkers of dementia, including phosphorylated tau (p-tau217), offer promise as tools for diagnosis, stratification for clinical trials, monitoring disease progression, and assessing the success of interventions in those living with Alzheimer’s disease. However, currently, it is unknown whether these dementia biomarker levels vary with the time of day, which could have implications for their clinical value. In two protocols, we studied 38 participants (70.8 ± 7.6 years; mean ± SD) in a 27-h laboratory protocol with either two samples taken 12 h apart or 3-hourly blood sampling for 24 h in the presence of a sleep–wake cycle. The study population comprised people living with mild Alzheimer’s disease (PLWA, *n* = 8), partners/caregivers of PLWA (*n* = 6) and cognitively intact older adults (*n* = 24). Single-molecule array technology was used to measure phosphorylated tau (p-tau217) (ALZpath), amyloid-beta 40 (Aβ40), amyloid-beta 42 (Aβ42), glial fibrillary acidic protein, and neurofilament light (NfL) (Neuro 4-Plex E). Analysis with a linear mixed model (SAS, PROC MIXED) revealed a significant effect of time of day for p-tau217, Aβ40, Aβ42, and NfL, and a significant effect of participant group for p-tau217. For p-tau217, the lowest levels were observed in the morning upon waking and the highest values in the afternoon/early evening. The magnitude of the diurnal variation for p-tau217 was similar to the reported increase in p-tau217 over one year in amyloid-β-positive mild cognitively impaired people. Currently, the factors driving this diurnal variation are unknown and could be related to sleep, circadian mechanisms, activity, posture, or meals. Overall, this work implies that the time of day of sample collection may be relevant in the implementation and interpretation of plasma biomarkers in dementia research and care.

## Introduction

Alzheimer’s disease (AD) is the most prevalent form of dementia, accounting for up to 80% of all cases, and hallmarks of the disease include amyloid plaques and hyperphosphorylated tau tangles in the brain [1, 2]. There is no standard approach to diagnose AD and disease presence cannot be determined by a single test but is rather a multi-faceted and multi-disciplinary approach involving taking medical history, cognitive tests, amyloid-PET scans, and sometimes cerebrospinal fluid (CSF) samples for measurement of amyloid-beta or tau [2]. However, imaging and CSF tests may not always be possible due to cost, availability of equipment, and the invasiveness of procedures which may not be well tolerated [3]. Nevertheless, the ability to confirm amyloid-beta pathology in the brain will become increasingly important with the advance of disease-modifying therapies (DMTs) targeting amyloidbeta e.g. the now discontinued aducanumab [4], lecanemab [5] and donanemab [6], as one of the requirements for prescribing these DMTs is confirmation of brain amyloid burden [7]. Thus, there is a need for acceptable, scalable, and accurate diagnostic approaches to determine disease presence, severity, and response to any treatment.

Plasma biomarkers offer an opportunity as a cost- and time-effective tool that is minimally invasive for screening and diagnosis, stratification, monitoring disease progression, and assessing treatment response. Biomarkers that have been proposed include amyloid-beta (Aβ40, Aβ42, and their ratio), phosphorylated tau (p-tau181 and p-tau217), glial fibrillary acidic protein (GFAP), and neurofilament light (NfL) (reviewed in [1]). The sensitivity and specificity of these biomarkers is an area of active research. In particular, p-tau217 has been demonstrated to be a valuable biomarker for predicting cognitive decline and monitoring treatment efficacy in response to DMT [8, 9].

Although plasma biomarkers, and particularly p-tau217 [10], show great promise as clinical tools very little is known about non-disease-related factors that may influence the concentrations of these biomarkers in blood. Biomarker levels may vary between individuals due to demographic or comorbid factors (inter-individual variation), but they may also vary within an individual due to behaviour or biological processes (intra-individual variation). Factors of interest include demographic variables such as age and sex, but also behavioural factors such as activity, posture, and eating and drinking. One factor of particular interest is the time of day since many physiological variables in blood display 24-h rhythmicity. However, to date, the impact of time-of-day has not been taken into consideration for the implementation of plasma biomarkers for dementia. The importance of time of day for diagnostic samples has already been demonstrated in other clinical conditions. For example, for people living with severe asthma, sputum samples from morning clinics have significantly higher levels of eosinophils than samples from afternoon clinics [11] which may impact clinical decision-making.

Here, we explored in a heterogenous group of participants consisting of people living with mild clinical Alzheimer’s disease (PLWA), their caregivers, and cognitively intact older adults, whether plasma levels of biomarkers of dementia-related brain changes over the course of a 24-h day. The data were collected under laboratory conditions that are similar to real-life conditions, i.e. in the presence of sleep–wake, dark–light cycle, and meals.

## Materials and methods

### Participants

#### Demographics

Data were collected from participants who were enroled in one of two studies: (1) in cognitively intact older adults and (2) in PLWA, their study partner, and cognitively intact older adults. The study protocol and eligibility criteria have previously been described in detail [12–14]. Briefly, eligibility was assessed using pre-defined inclusion/exclusion criteria for each of the three study groups. PLWA had to be 50–85 years old with a confirmed diagnosis of prodromal or mild clinical AD, have an SMMSE (standardised mini-mental state examination (MMSE) [15]) score ≥ 23, be living in the community and be on a stable dose of any medication for dementia for at least three months. The diagnosis of prodromal or mild AD was based on clinical history, cognitive tests, and CT/MRI imaging. PLWA could participate in the study by themselves, or they could have a ‘study partner’ who must have known them for at least six months and could be their carer or a family member or friend. Study partners were ≥18 years old and had to have an SMMSE score ≥ 27. Cognitively intact older adults had to be aged 50–85 years, have an SMMSE score ≥ 27 (Study Two), and any comorbidities and concomitant medications must have been stable for the past three months. Cognitively intact adults were recruited via the Surrey Clinical Research Facility database. Potentially eligible PLWA and their study partners were identified via Surrey and Borders Partnership NHS Foundation Trust (SABP) memory services and were approached by one of the SABP team initially by telephone to discuss the study before being provided with the participant information sheet.

#### Ethics approval and consent to participate

Study One (cognitively intact older adults) received a favourable opinion from the University of Surrey Ethics Committee (UEC 2019 065 FHMS), and Study Two (PLWA, caregivers of PLWA, and cognitively intact older adults) received a favourable opinion from an NHS ethics committee (London—City & East Research Ethics Committee: 22/LO/0694). Study Two is registered as a clinical study on the ISRCTN (International Standard Randomised Controlled Trial Number) registry (ISRCTN10509121). The protocols were conducted in accordance with the Declaration of Helsinki and guided by the principles of Good Clinical Practice. All personal data were handled in accordance with the General Data Protection Regulations and the UK Data Protection Act 2018. Written informed consent was obtained from participants prior to any study procedures being performed. Participants were compensated for their time and inconvenience.

### Procedures and measures

The full study protocols have been reported in detail elsewhere [12, 13]. Briefly, following a screening visit to assess eligibility, participants were monitored for up to 14 days at home using a variety of technologies to assess their sleep–wake patterns, environment, and cognitive function. They then attended the UK-DRI Clinical Research Facility at the University of Surrey for a 27-h residential session which included a full clinical polysomnography (PSG) recording during an extended 10-h period in bed. PSG was recorded using the Somnomedics SomnoHD system with Domino software (v 3.0.0.6; sampled at 256 Hz; SOMNOmedics GmbHTM, Germany) with an American Academy of Sleep Medicine standard adult montage. Habitual bedtime was determined from the information provided in the Pittsburgh Sleep Quality Index (PSQI) [14] and the PSG metrics have been previously reported for Study 1 [12].

During the residential session, participants remained in environmentally controlled bedroom environments with en-suite facilities. For PLWA and their study partners, the aim was to recreate their sleeping situation at home so they could either share a room in a double occupancy suite or be in adjacent rooms with an interconnecting door. During the afternoon/evening/morning hours, participants were free to pursue their own activities around scheduled procedures including sample collection, meals, questionnaire completion, and having PSG equipment attached.

#### Study one

Participants had two blood samples drawn 12 h apart via venepuncture at 19:46 ± 00:33 h and 07:53 ± 00:35 h (mean ± SD). The evening sample was 2.88 ± 0.80 h before lights off, and the morning sample was 0.68 ± 0.92 h after lights on. Dinner was scheduled ~5 h before habitual bedtime (at approximately 18:30 h) and breakfast ~2 h after habitual waking (at approximately 09:30 h). Thus, the evening sample was taken after dinner and the morning sample before breakfast.

#### Study two

Participants had an indwelling cannula sited and blood samples were drawn at three-hourly intervals relative to their habitual bedtime. Sampling began 9 h before habitual bedtime and continued until 15 h after habitual bedtime. Lunch was ~9.5 h and dinner was ~4 h before habitual bedtime; breakfast was ~1.5 h after habitual waketime. Meals varied in content due to individual preference, but the relative sizes of each meal were consistent between individuals.

Blood samples were collected into K2 EDTA Vacuettes which were centrifuged within 10 min of collection at 4 °C at 1620 × *g* for 10 min. The plasma fraction was separated and stored at −80 °C. Samples were shipped to the UK DRI Biomarker Factory, UCL, London where they were analysed using Simoa HD-X technology. The following biomarkers were measured in both studies using the Neuro 4-PlexE assay kit (Quanterix, Billerica MA): amyloid-beta 40 (Aβ40), amyloid-beta 42 (Aβ42), GFAP, and NfL. Tau phosphorylated at threonine 217 (p-tau217) was measured using the ALZpath Simoa assay and measured in Study Two only (ALZpath, Carlsbad, CA). Samples were measured blind in singlicate, and four internal controls made of pooled plasma were used to monitor any intra-and inter-plate variation. All coefficients of variation for internal assay controls were below 10%.

### Data analysis

For each biomarker, the mean values at each timepoint were computed and also intraclass correlations (ICCs) were calculated using R Statistical Software (v4.2.2; R Core Team 2022).

To assess time of day effects we created two analysis sets. Analysis set 1: we combined the evening and morning samples from study 1 and study 2 which allowed for a comparison of evening vs morning samples. For study 2, we used the samples 3 h before and 9 h after habitual bedtime. Analysis set 2: this consisted of the samples collected at the 9 time points in study 2. For both analysis sets, a PROC MIXED linear model (SAS v9.4, SAS Institute Inc) was run in which participant was the random effect with factors time-of-day, group, and their interaction. A second PROC MIXED linear model was run on the two analysis sets to investigate any effects of covariates: age, sex, BMI, PSQI, and PSG apnoea–hypopnea index (AHI) in addition to the effects of time of day and group.

## Results

Here we report data from 38 participants (Supplementary Table 1) whose comorbidities included hypertension, Type-2 diabetes, arthritis, hyperthyroidism, and asthma [12, 13]

For both studies combined, 90% of scheduled samples were obtained. For the nine timepoint comparisons (Study Two), the plasma levels for each biomarker at each timepoint (mean ± SD) for all participants combined, as well as separately for each group are presented in Table 1. The ICCs for all participants combined ranged between 0.84 and 0.97 for the different biomarkers and the ICC values were similar across groups. Table 2 provides a similar comparison for the two-time points (evening vs morning) comparison, and here the ICCs range from 0.76 to 0.93 for all participants combined. These ICC values imply that the between-participant variation is greater than the within-participant variation and that this is similar across groups.

**Table 1:** Plasma biomarker levels (pg/mL, mean ± SD) across a 24-h period.

Variable	Group	Statistics	Time-point (approximate clock time)									ICC, (95% CI)
			1 (14:00)	2 (17:00)	3 (20:00)	4 (23:00)	5 (02:00)	6 (05:00)	7 (08:00)	8 (11:00)	9 (14:00)	
P-tau217	Total sample	Mean	0.789	0.686	0.767	0.671	0.674	0.670	0.653	0.612	0.654	0.97 (0.96, 0.99)
		SD	0.593	0.501	0.676	0.481	0.498	0.514	0.519	0.472	0.496	
		*N*	20	20	21	17	17	17	20	17	17	
	Cognitively intact	Mean	0.361	0.378	0.348	0.346	0.341	0.337	0.332	0.327	0.385	0.89 (0.74, 0.98)
		SD	0.118	0.091	0.129	0.094	0.101	0.128	0.114	0.121	0.205	
		*N*	7	7	7	7	7	7	7	7	6	
	PLWA	Mean	1.272	1.127	1.323	1.248	1.297	1.294	1.144	1.129	1.121	0.95 (0.89, 0.99)
		SD	0.660	0.599	0.810	0.440	0.420	0.470	0.591	0.564	0.565	
		*N*	8	7	8	5	5	5	7	5	6	
	Study partner	Mean	0.615	0.530	0.515	0.550	0.518	0.511	0.455	0.494	0.415	0.94 (0.85, 0.99)
		SD	0.245	0.280	0.269	0.317	0.301	0.312	0.243	0.237	0.164	
		*N*	5	6	6	5	5	5	6	5	5	
Aβ40	Total sample	Mean	122.420	119.280	123.916	125.762	129.257	123.698	118.405	111.887	124.890	0.86 (0.77, 0.93)
		SD	26.665	24.698	30.104	26.949	27.169	23.497	23.771	26.566	22.899	
		*N*	20	20	21	17	17	17	20	17	17	
	Cognitively intact	Mean	117.884	118.666	121.981	128.279	128.248	124.966	114.608	106.928	124.004	0.87 (0.71, 0.97)
		SD	32.883	30.941	33.746	27.547	32.163	26.646	26.635	25.072	26.415	
		*N*	7	7	7	7	7	7	7	7	6	
	PLWA	Mean	131.905	124.608	134.932	127.641	138.823	134.975	129.656	120.552	125.563	0.84 (0.67, 0.96)
		SD	22.686	19.975	31.770	24.622	24.064	18.901	20.061	29.313	24.201	
		*N*	8	7	8	5	5	5	7	5	6	
	Study partner	Mean	113.597	113.781	111.484	120.360	121.104	110.645	109.710	110.166	125.144	0.88 (0.72, 0.98)
		SD	23.273	24.776	21.460	33.294	24.953	20.281	22.910	29.671	22.164	
		*N*	5	6	6	5	5	5	6	5	5	
Aβ42	Total sample	Mean	7.941	8.015	8.110	8.370	8.575	8.294	7.884	7.342	8.134	0.84 (0.74, 0.92)
		SD	1.645	1.595	1.884	1.774	1.714	1.559	1.699	1.465	1.547	
		*N*	20	20	21	17	17	17	20	17	17	
	Cognitively intact	Mean	8.303	8.477	8.591	9.192	9.299	9.111	8.276	7.769	8.769	0.83 (0.65, 0.96)
		SD	2.036	2.060	2.448	1.699	1.994	1.886	2.038	1.514	1.607	
		*N*	7	7	7	7	7	7	7	7	6	
	PLWA	Mean	8.105	8.006	8.475	7.646	7.934	7.847	8.162	6.891	7.801	0.86 (0.7, 0.96)
		SD	1.517	1.310	1.643	1.666	1.382	0.935	1.527	1.382	1.516	
		*N*	8	7	8	5	5	5	7	5	6	
	Study partner	Mean	7.171	7.488	7.062	7.943	8.202	7.596	7.104	7.194	7.773	0.82 (0.61, 0.97)
		SD	1.257	1.372	1.171	1.850	1.507	1.222	1.458	1.623	1.594	
		*N*	5	6	6	5	5	5	6	5	5	
Aβ42/Aβ40	Total sample	Mean	0.066	0.068	0.066	0.067	0.067	0.068	0.067	0.067	0.066	0.92 (0.87, 0.96)
		SD	0.011	0.011	0.010	0.009	0.009	0.009	0.009	0.011	0.010	
		*N*	20	20	21	17	17	17	20	17	17	
	Cognitively intact	Mean	0.071	0.072	0.071	0.072	0.073	0.073	0.072	0.074	0.071	0.9 (0.76, 0.98)
		SD	0.011	0.011	0.008	0.010	0.007	0.009	0.008	0.011	0.006	
		*N*	7	7	7	7	7	7	7	7	6	
	PLWA	Mean	0.062	0.065	0.064	0.060	0.057	0.059	0.063	0.058	0.063	0.95 (0.88, 0.99)
		SD	0.012	0.014	0.013	0.008	0.005	0.008	0.010	0.010	0.014	
		*N*	8	7	8	5	5	5	7	5	6	
	Study partner	Mean	0.064	0.067	0.064	0.067	0.068	0.069	0.065	0.066	0.062	0.84 (0.63, 0.97)
		SD	0.005	0.007	0.006	0.006	0.004	0.004	0.007	0.006	0.006	
		*N*	5	6	6	5	5	5	6	5	5	
GFAP	Total sample	Mean	147.624	140.967	148.273	130.534	143.213	140.273	142.536	123.011	136.349	0.9 (0.83, 0.95)
		SD	77.600	68.563	89.029	71.707	75.650	70.634	68.234	63.581	69.134	
		*N*	20	20	21	17	17	17	20	17	17	
	Cognitively intact	Mean	98.161	105.159	97.122	95.467	109.904	111.400	117.094	97.228	115.692	0.73 (0.49, 0.93)
		SD	25.105	26.680	35.601	27.099	37.948	47.691	47.438	35.906	49.709	
		*N*	7	7	7	7	7	7	7	7	6	
	PLWA	Mean	196.745	185.026	204.898	180.384	195.483	197.520	182.919	166.679	177.503	0.86 (0.7, 0.96)
		SD	90.111	80.013	99.056	75.704	93.266	74.099	76.113	71.305	75.510	
		*N*	8	7	8	5	5	5	7	5	6	
	Study partner	Mean	138.279	131.342	132.451	129.779	137.577	123.448	125.105	115.437	111.753	0.94 (0.85, 0.99)
		SD	65.049	68.577	85.748	92.065	81.464	71.730	66.979	74.387	70.464	
		*N*	5	6	6	5	5	5	6	5	5	
NfL	Total sample	Mean	23.415	21.750	22.150	22.279	23.550	23.821	22.445	21.413	21.031	0,97 (0.94, 0.98)
		SD	11.023	10.870	12.087	11.539	11.638	10.991	11.922	11.171	11.601	
		*N*	20	20	21	17	17	17	20	17	17	
	Cognitively intact	Mean	17.935	17.141	16.552	17.117	17.804	18.529	17.490	17.069	17.752	0.93 (0.83, 0.98)
		SD	7.012	5.757	7.056	6.707	5.677	6.123	6.638	5.751	7.422	
		*N*	7	7	7	7	7	7	7	7	6	
	PLWA	Mean	26.948	25.942	27.439	25.279	28.854	29.074	27.455	23.782	24.570	0.87 (0.73, 0.97)
		SD	6.569	5.243	7.881	3.200	6.131	4.028	7.119	5.320	4.383	
		*N*	8	7	8	5	5	5	7	5	6	
	Study partner	Mean	25.433	22.237	21.629	26.508	26.291	25.976	22.379	25.125	20.719	0.99 (0.97, 1)
		SD	18.582	17.933	18.676	19.356	18.801	17.930	18.892	19.079	20.258	
		*N*	5	6	6	5	5	5	6	5	5

**Table 2:** Plasma biomarker levels (pg/mL, mean ± SD): evening vs morning.

Variable	Group	Time point	Mean	SD	ICC, (95% CI)
P-tau217 (*N* = 21)	Total sample	p.m.	0.767	0.676	0.93 (0.83, 0.97)
		a.m.	0.653	0.519	
	Cognitively intact	p.m.	0.348	0.129	0.94 (0.72, 0.99)
		a.m.	0.332	0.114	
	PLWA	p.m.	1.323	0.810	0.86 (0.5, 0.97)
		a.m.	1.144	0.591	
	Study partner	p.m.	0.515	0.269	0.95 (0.74, 0.99)
		a.m.	0.455	0.243	
Aβ40 (*N* = 38)	Total sample	p.m.	126.617	27.178	0.76 (0.58, 0.86)
		a.m.	120.833	22.196	
	Cognitively intact	p.m.	127.628	26.383	0.67 (0.38, 0.84)
		a.m.	121.050	22.276	
	PLWA	p.m.	134.932	31.770	0.84 (0.44, 0.97)
		a.m.	129.656	20.061	
	Study partner	p.m.	111.484	21.460	0.97 (0.82, 1)
		a.m.	109.710	22.910	
Aβ42 (*N* = 38)	Total sample	p.m.	7.898	1.907	0.88 (0.78, 0.93)
		a.m.	7.722	1.667	
	Cognitively intact	p.m.	7.914	2.106	0.87 (0.73, 0.94)
		a.m.	7.750	1.771	
	PLWA	p.m.	8.475	1.643	0.89 (0.58, 0.98)
		a.m.	8.162	1.527	
	Study partner	p.m.	7.062	1.171	0.88 (0.44, 0.98)
		a.m.	7.104	1.458	
Aβ42/Aβ40 (*N* = 38)	Total sample	p.m.	0.063	0.011	0.91 (0.83, 0.95)
		a.m.	0.064	0.010	
	Cognitively intact	p.m.	0.062	0.012	0.91 (0.8, 0.96)
		a.m.	0.064	0.011	
	PLWA	p.m.	0.064	0.013	0.95 (0.78, 0.99)
		a.m.	0.063	0.010	
	Study partner	p.m.	0.064	0.006	0.82 (0.25, 0.97)
		a.m.	0.065	0.007	
GFAP (*N* = 38)	Total sample	p.m.	148.756	77.933	0.83 (0.7, 0.91)
		a.m.	145.723	65.281	
	Cognitively intact	p.m.	134.118	61.617	0.87 (0.72, 0.94)
		a.m.	139.782	59.893	
	PLWA	p.m.	204.898	99.056	0.69 (0.09, 0.93)
		a.m.	182.919	76.113	
	Study partner	p.m.	132.451	85.748	0.95 (0.75, 0.99)
		a.m.	125.105	66.979	
NfL (*N* = 38)	Total sample	p.m.	22.500	9.626	0.93 (0.87, 0.96)
		a.m.	23.344	9.679	
	Cognitively intact	p.m.	21.072	6.597	0.84 (0.66, 0.93)
		a.m.	22.345	6.887	
	PLWA	p.m.	27.439	7.881	0.87 (0.52, 0.97)
		a.m.	27.455	7.119	
	Study partner	p.m.	21.629	18.676	0.99 (0.97, 1)
		a.m.	22.379	18.892	

**Fig. 1:**
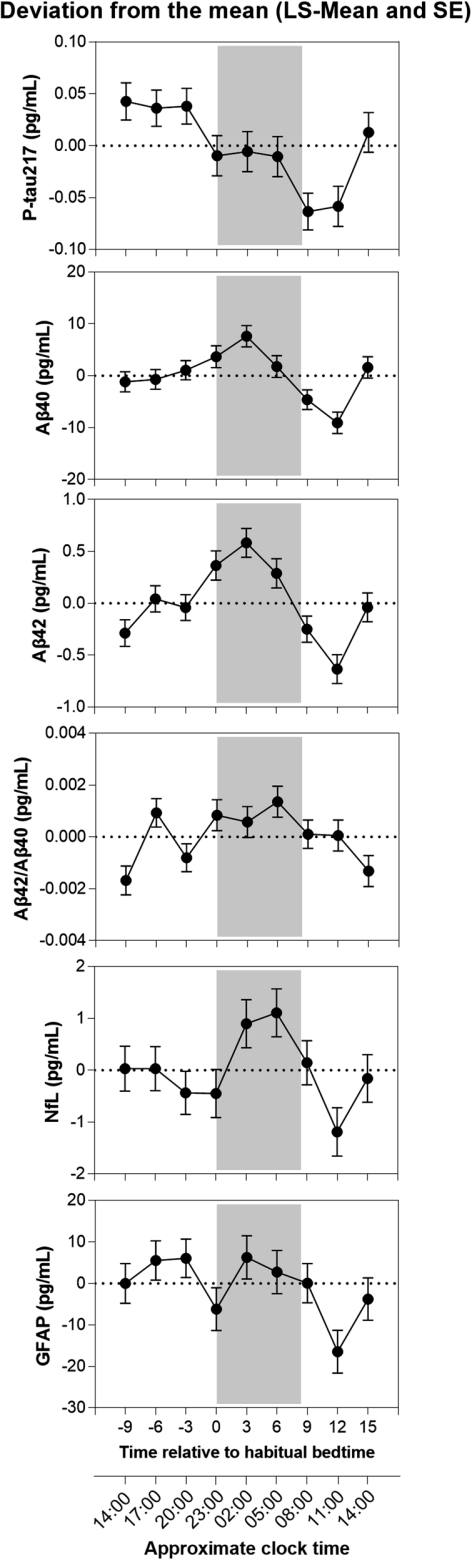
Levels of plasma biomarkers (deviation from the mean LS-means ± SE) across a 24-h period: p-tau217, Aβ40, Aβ42, Aβ42/Aβ40, NfL, and GFAP. The grey shading indicates the habitual sleep episode.

For the Study 2 dataset, with nine-time points, the model showed that there was a significant main effect of time for all biomarkers (*p* < 0.01) except GFAP (*p* = 0.065) (Table 3). Figure 1 shows plasma biomarker levels (LS means for the deviation from the mean) for all participants across 24-h. For plasma p-tau217 (LS-means) lowest values were observed in the morning, and shortly after wake time, after which levels rose to the highest values in the afternoon and evening. Thus, p-tau217 concentrations in the first two samples after wake time were significantly lower (*p* < 0.0001) compared to the evening (3 h before habitual bedtime) sample. For Aβ40, Aβ42 and NfL, peak levels occurred during the sleep episode and lowest levels in the morning hours. The magnitude of the diurnal variation (change in LS-means expressed as a percentage from the overall mean) was: 14.0% (Aβ40), 15.3% (Aβ42), 4.6% (Aβ42/ Aβ40), 10.6% (NfL), 17.0% (GFAP), and 15.8% (p-tau217).

**Table 3:** Summary of PROC MIXED analysis for plasma biomarkers over nine-time points.

			Main effect, (*N* = 21)			
Variable	Time		Group		Time × group	
	*F* (DF)	*p*	*F* (DF)	*p*	*F* (DF)	*p*
P-tau217	4.29 (8, 121)	**0.0001**	8.57 (2, 17.9)	**0.003**	1.28 (16, 121)	0.219
Aβ40	4.78 (8, 121)	**<0.0001**	0.72 (2, 17.9)	0.501	0.92 (16, 121)	0.549
Aβ42	6.36 (8, 121)	**<0.0001**	0.98 (2, 17.9)	0.393	0.66 (16, 121)	0.824
Aβ42/Aβ40	3 (8, 121)	**0.0042**	1.46 (2, 18)	0.259	1 (16, 121)	0.466
GFAP	1.91 (8, 121)	0.065	3.1 (2, 18)	0.069	1.26 (16, 121)	0.232
NfL	2.05 (8, 121)	**0.046**	1.41 (2, 18)	0.270	0.91 (16, 121)	0.558

Bold values are statistically significant (*p* < 0.05).

**Fig. 2:**
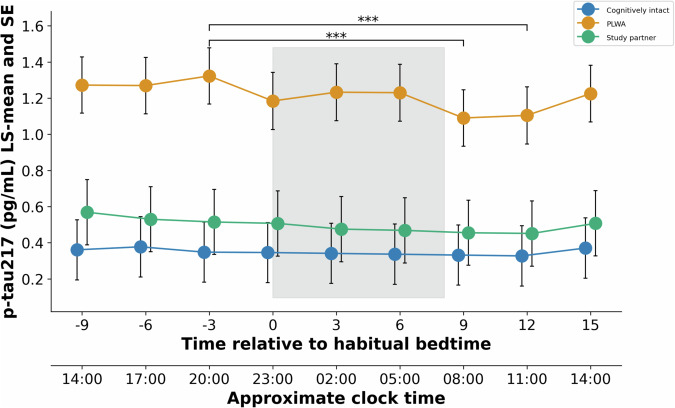
Levels of plasma p-tau217 (LS-means ± SE) across a 24-h period in PLWA, their study partners, and cognitively intact older adults. Blue symbols represent cognitively intact older adults, green symbols represent study partners, and orange symbols represent PLWA. The grey shading indicates the habitual sleep episode. ***Indicates a significant (*p* < 0.0001) difference in levels between the indicated time points in PLWA. The data for the Study partners and the cognitively intact older adults are displaced by 15 min so that the variance indicators of the various groups are visible.

A significant effect of the group was observed for p-tau217 (*p* = 0.003) (Fig. 2) with the highest levels observed in PLWA, and the effect of the group approached significance for GFAP (*p* = 0.069). A significant group-by-time interaction was not observed for any of the biomarkers. For p-tau217, the magnitude of the diurnal variation in PLWA estimated from the LS-means (0.233 ± 0.044, LS-mean ± SE) was 27% of the difference between the mean values for cognitively intact adults (0.349 ± 0.164, LS-mean ± SE) and PLWA (1.215 ± 0.153, LS-mean ± SE).

To further establish the effects of time we compared evening to morning samples using data from both studies 1 and 2. In this comparison, data were available for 38 participants for all biomarkers except p-tau217 (*n* = 21). PROC MIXED analysis on the two-time points only (Table 4) revealed significant effects of time, group, and group-by-time interaction for p-tau217 only.

**Table 4:** Summary of PROC MIXED analysis for plasma biomarkers over two-time points.

				Main effect			
Variable	*N*	Time		Group		Time × group	
		*F* (DF)	*p*	*F* (DF)	*P*	*F* (DF)	*p*
P-tau217	21	7.77 (1, 17)	**0.013**	7.35 (2, 17.9)	**0.005**	3.66 (2, 17)	**0.048**
Aβ40	38	3.86 (1, 32.6)	0.058	1.19 (2, 34.2)	0.315	0.42 (2, 32.6)	0.662
Aβ42	38	1.89 (1, 32.9)	0.179	0.73 (2, 34.7)	0.488	0.54 (1, 32.9)	0.5869
Aβ42/Aβ40	38	1.38 (1, 33.1)	0.248	0.09 (2, 35)	0.914	0.18 (2, 331.1)	0.839
GFAP	38	2.9 (1, 33)	0.098	1.9 (2, 34.8)	0.165	2.87 (2, 33)	0.071
NfL	38	0.27 (1, 33.2)	0.606	1.03 (2, 35.1)	0.367	0.72 (2, 33.2)	0.495

Bold values are statistically significant (*p* < 0.05).

When the covariates (age, sex, BMI, PSQI, PSG-AHI) were added to the model and applied to the nine-time points, the effects of time remained significant for p-tau217, Aβ40, Aβ42, Aβ42/ Aβ40, and NfL. For p-tau217 the effect of group remained significant and a significant interaction between time and group emerged. A significant effect of age was observed for GFAP (*p* = 0.036). No significant effects of sex, BMI, PSQI or PSG-AHI were observed for any of the biomarkers (Supplementary Table 2). For the two timepoint datasets (Supplementary Table 3), a similarly significant effect of age was observed for GFAP (*p* = 0.006) and for p-tau217 the significant effects of time, group, and group*time remained.

## Discussion

Here, we show that levels of commonly used plasma biomarkers in dementia research including p-tau217, Aβ40, Aβ42, Aβ42/Aβ40, and NfL vary with time of day. This significant variation with time-of-day was observed despite the rather large ICC values (range 0.76–0.97), which indicate that the between-participant variation is greater than the within-participant variation. The ICCs reported here are in line with previous studies which investigated the longitudinal reliability of plasma biomarkers and observed ICC values between 0.66 and 0.78 [16]. Our observed impact of age on GFAP levels is in line with previous observations in people living with Parkinson’s disease where GFAP was shown to correlate with both age and MMSE [17].

The mean values of p-tau217 observed ranged between 0.32 and 0.62 pg/mL for cognitively intact participants and study partners, and between 1.1 pg/mL and 1.4 pg/mL for PLWA. These ranges are in line with those previously reported where <0.40 pg/mL indicated a negative p-tau217 result and >0.63 pg/mL a positive result [18].

We observed significant time-of-day variation for p-tau217, NfL, Aβ40, Aβ42, and Aβ42/Aβ40 with the effect approaching significance for GFAP, with the magnitude of diurnal variation ranging from 4.6% to 15.8% for the significant effects. Previous work has demonstrated that cerebrospinal (CSF) levels of amyloid-beta fluctuate with time of day [19–21]. The observed diurnal fluctuations for Aβ40 and Aβ42 were 2.6% and 0.4%, respectively, for amyloid-positive participants, with the highest values in the early afternoon and lowest values upon waking [20]. This compares to a 14.0% for Aβ40 and 15.3% for Aβ42 diurnal variation in plasma observed in the current study.

The exact shape of the diurnal variation varied across the biomarkers, but the lowest values were in general observed in the morning. For p-tau217 highest values were observed before bedtime and the lowest values upon awakening. For Aβ40 and Aβ42, we observed the highest values during the nocturnal sleep period and the lowest values upon waking. A previous study of Aβ40, and Aβ42 in CSF showed levels were lower in the morning with the highest values in the afternoon [20]. For NfL, the highest values were also observed during the sleep period with the lowest values in mid-morning and relatively stable levels in the afternoon/evening and morning. Larger sample sizes are needed to further determine the precise shape of this diurnal variation and differences therein across the biomarkers.

The factors underlying the observed diurnal variation remain to be identified. They could be related to circadian modulation of production, phosphorylation, and clearance from the brain or could be a response to behavioural changes/processes across the 24-h day including sleep, meals, or posture. In the latter case, simple behavioural constraints could remove the variance and samples could be taken at any time, whereas in the former case, samples should be taken within particular time windows or values should be corrected for the time of day. The observed differences in the shape and timing of the diurnal variation across the biomarkers make it unlikely that one common mechanism, such as changes in blood volume, or circadian or sleep-mediated clearance from the brain into the circulation drives all of this diurnal variation.

Although the time-of-day effects we observed may appear small, when they are placed in the context of disease or treatment monitoring, they become of clinical interest.

For example, plasma p-tau217 has recently become a biomarker of interest in AD research due to its sensitivity for discriminating for AD, its ability to predict cognitive decline, and its capacity to track response to DMT [8, 9, 22]. Of particular interest is a study in cohorts of Aβ positive individuals (*n* = 171) who were cognitively unimpaired [8]. In this study, cognition was assessed using the MMSE and the modified preclinical Alzheimer Cognitive Composite (mPACC) over a median of six years. Plasma p-tau217 was shown to be the strongest biomarker for predicting cognitive decline and also conversion to AD [8]. Of particular relevance to our findings is that longitudinal monitoring in those with Aβ-positive prodromal AD showed an increase in p-tau217 of 14.7% per year [23]. This is very similar to the magnitude of the diurnal variation (15.8%) observed in the current study. This change is also meaningful when we consider that in the TRAILBLAZER-ALZ clinical trial following treatment with donanemab for up to 72 weeks, plasma-tau217 levels declined by 23% [9] and GFAP levels decreased by 12%, whereas under placebo both biomarkers increased by 6% and 15%. These percentages are also within the range of the systematic effect of time of day observed in our study.

Of the plasma biomarkers assessed in the TRAILBLAZER Trial (GFAP, NfL, p-tau217, and Aβ42/Aβ40) only p-tau217 was positively and significantly associated with baseline amyloid plaques and global tau deposition. It is of interest that in our small sample only p-tau217 showed a significant group effect.

For now, our results suggest that time of day matters when considering sampling for plasma biomarkers of dementia for monitoring disease progression or treatment outcome. This time-of-day variation was observed despite the presence of confounding factors that would be present in the real world including a light/dark cycle, sleep/wake state, and meals. As such, samples obtained at an early morning clinic may provide different results to those taken in an afternoon or evening clinic. Time of day should be standardised or at least recorded when samples are collected whether for diagnosis or monitoring their clinical status longitudinally. Recent studies suggest that biomarker concentrations also vary by food intake [24]. For now, we recommend that reference limits for biomarkers related to neurodegenerative dementias are established in samples collected while fasting and in the morning, and that samples for dementia diagnostics are collected accordingly.

## Supplementary information

Supplementary tables

## Data Availability

The datasets generated and analysed during this study are available from the author CdM on reasonable request.

## References

[1] Blennow K, Galasko D, Perneczky R, Quevenco FC, van der Flier WM, Akinwonmi A, et al. The potential clinical value of plasma biomarkers in Alzheimer’s disease. Alzheimers Dement. 2023;19:5805–16. 37694991 10.1002/alz.13455

[2] Alzheimer's Association Report. 2020 Alzheimeras disease facts and figures. Alzheimers Dement. 2020;16:391–460. 10.1002/alz.12068

[3] Bittner T. Editorial: What are the remaining challenges before blood-based biomarkers for Alzheimer’s disease can be used in clinical practice? J Prev Alzheimers Dis. 2022;9:567–8. 36281660 10.14283/jpad.2022.89

[4] Budd Haeberlein S, Aisen PS, Barkhof F, Chalkias S, Chen T, Cohen S, et al. Two randomized phase 3 studies of aducanumab in early Alzheimer’s disease. J Prev Alzheimers Dis. 2022;9:197–210. 35542991 10.14283/jpad.2022.30

[5] van Dyck CH, Swanson CJ, Aisen P, Bateman RJ, Chen C, Gee M, et al. Lecanemab in early Alzheimer’s disease. N Engl J Med. 2023;388:9–21. 36449413 10.1056/NEJMoa2212948

[6] Sims JR, Zimmer JA, Evans CD, Lu M, Ardayfio P, Sparks J, et al. Donanemab in early symptomatic Alzheimer disease: the TRAILBLAZER-ALZ 2 randomized clinical trial. JAMA. 2023;330:512–27. 37459141 10.1001/jama.2023.13239 PMC10352931

[7] Cummings J, Aisen P, Apostolova LG, Atri A, Salloway S, Weiner M. Aducanumab: appropriate use recommendations. J Prev Alzheimers Dis. 2021;8:398–410. 34585212 10.14283/jpad.2021.41 PMC8835345

[8] Mattsson-Carlgren N, Salvado G, Ashton NJ, Tideman P, Stomrud E, Zetterberg H, et al. Prediction of longitudinal cognitive decline in preclinical Alzheimer disease using plasma biomarkers. JAMA Neurol. 2023;80:360–9. 36745413 10.1001/jamaneurol.2022.5272 PMC10087054

[9] Pontecorvo MJ, Lu M, Burnham SC, Schade AE, Dage JL, Shcherbinin S, et al. Association of donanemab treatment with exploratory plasma biomarkers in early symptomatic Alzheimer disease: a secondary analysis of the TRAILBLAZER-ALZ randomized clinical trial. JAMA Neurol. 2022;79:1250–9. 36251300 10.1001/jamaneurol.2022.3392 PMC9577883

[10] Woo MS, Tissot C, Lantero-Rodriguez J, Snellman A, Therriault J, Rahmouni N, et al. Plasma pTau-217 and N-terminal tau (NTA) enhance sensitivity to identify tau PET positivity in amyloid-beta positive individuals. Alzheimers Dement. 2024;20:1166–74. 37920945 10.1002/alz.13528 PMC10916953

[11] Durrington HJ, Gioan-Tavernier GO, Maidstone RJ, Krakowiak K, Loudon ASI, Blaikley JF, et al. Time of day affects eosinophil biomarkers in asthma: implications for diagnosis and treatment. Am J Respir Crit Care Med. 2018;198:1578–81. 30156881 10.1164/rccm.201807-1289LE PMC6298638

[12] Ravindran GKK, della Monica C, Atzori G, Lambert D, Hassanin H, Revell V, et al. Three contactless sleep technologies compared to actigraphy and polysomnography in a heterogenous group of older men and women in a model of mild sleep disturbance: a sleep laboratory study. JMIR Mhealth Uhealth. 2023;11:46338. 10.2196/46338 PMC10632916 37878360

[13] Ravindran KKG, della Monica C, Atzori G, Lambert D, Hassanin H, Revell V, et al. Contactless and longitudinal monitoring of nocturnal sleep and daytime naps in older men and women: a digital health technology evaluation study. Sleep. 2023;46:zsad194. 10.1093/sleep/zsad194 PMC10566241 37471049

[14] della Monica C, Ravindran KK, Atzori G, Lambert DJ, Rodriguez T, Mahvash-Mohammadi S, et al. A protocol for evaluating digital technology for monitoring sleep and circadian rhythms in older people and people living with dementia in the community. Clocks Sleep. 2024;6:129–55. 38534798 10.3390/clockssleep6010010 PMC10968838

[15] Molloy DW, Standish TI. A guide to the standardized mini-mental state examination. Int Psychogeriatr. 1997;9:87–94. 9447431 10.1017/S1041610297004754

[16] Bilgel M, An Y, Walker KA, Moghekar AR, Ashton NJ, Kac PR, et al. Longitudinal changes in Alzheimer’s-related plasma biomarkers and brain amyloid. Alzheimers Dement. 2023;19:4335–45. 37216632 10.1002/alz.13157 PMC10592628

[17] Tang Y, Han L, Li S, Hu T, Xu Z, Fan Y, et al. Plasma GFAP in Parkinson’s disease with cognitive impairment and its potential to predict conversion to dementia. NPJ Parkinsons Dis. 2023;9:23. 36759508 10.1038/s41531-023-00447-7 PMC9911758

[18] Ashton NJ, Brum WS, Di Molfetta G, Benedet AL, Arslan B, Jonatis E, et al. Diagnostic accuracy of the plasma ALZpath pTau217 immunoassay to identify Alzheimer’s disease pathology. medRxiv: 2023.07.11.23292493 [Preprint]. 2023 [cited 2023 Nov 7]. Available from: 10.1101/2023.07.11.23292493

[19] Bateman RJ, Wen G, Morris JC, Holtzman DM. Fluctuations of CSF amyloid-beta levels: implications for a diagnostic and therapeutic biomarker. Neurology. 2007;68:666–9. 17325273 10.1212/01.wnl.0000256043.50901.e3

[20] Dobrowolska JA, Kasten T, Huang Y, Benzinger TL, Sigurdson W, Ovod V, et al. Diurnal patterns of soluble amyloid precursor protein metabolites in the human central nervous system. PLoS One. 2014;9:e89998. 24646516 10.1371/journal.pone.0089998 PMC3960093

[21] Lucey BP, Fagan AM, Holtzman DM, Morris JC, Bateman RJ. Diurnal oscillation of CSF Abeta and other AD biomarkers. Mol Neurodegener. 2017;12:36. 28478762 10.1186/s13024-017-0161-4 PMC5421331

[22] Palmqvist S, Janelidze S, Quiroz YT, Zetterberg H, Lopera F, Stomrud E, et al. Discriminative accuracy of plasma phospho-tau217 for Alzheimer disease vs other neurodegenerative disorders. JAMA. 2020;324:772–81. 32722745 10.1001/jama.2020.12134 PMC7388060

[23] Mattsson-Carlgren N, Janelidze S, Palmqvist S, Cullen N, Svenningsson AL, Strandberg O, et al. Longitudinal plasma p-tau217 is increased in early stages of Alzheimer’s disease. Brain. 2020;143:3234–41. 33068398 10.1093/brain/awaa286 PMC7719022

[24] Huber H, Ashton NJ, Schieren A, Montoliu-Gaya L, Molfetta GD, Brum WS, et al. Levels of Alzheimer’s disease blood biomarkers are altered after food intake—a pilot intervention study in healthy adults. Alzheimers Dement. 2023;19:5531–40. 37243891 10.1002/alz.13163

