# Supplementary material for: P-tau217 and other blood biomarkers of dementia: variation with time of day
Source: Transl Psychiatry. 2024 Sep 13;14:373. doi: 10.1038/s41398-024-03084-7 (PMC11399374; doi:10.1038/s41398-024-03084-7)
Supplement: Supplementary file 1 — Supplementary tables [file 41398_2024_3084_MOESM1_ESM.docx]

Translational Psychiatry. P-tau217 and other blood biomarkers of dementia: variation with time of day.

**Supplementary tables**

**Supplementary Table 1. Participant demographic information.**

|  | **N** | **Males** | **Females** | **Age** | | **MMSE** | | **BMI** | |
| --- | --- | --- | --- | --- | --- | --- | --- | --- | --- |
|  |  |  |  | ***Mean*** | ***SD*** | ***Mean*** | ***SD*** | ***Mean*** | ***SD*** |
| Cognitively Intact | 24 | 15 | 9 | 70.5 | 5.4 | 28.9 | 1.2 | 26.4 | 4.4 |
| PLWA | 8 | 4 | 4 | 74.8 | 4.4 | 27.0 | 1.8 | 29.7 | 7.6 |
| Study partner | 6 | 3 | 3 | 66.7 | 14.8 | 28.8 | 1.0 | 28.9 | 4.15 |

**Supplementary Table 2. Mixed model results including age, sex, BMI, PSQI, and AHI in the model for the nine-time points.**

|  | **Main effect (N = 21)** | | | | | | | | | | | | | | | | | | | | | | |
| --- | --- | --- | --- | --- | --- | --- | --- | --- | --- | --- | --- | --- | --- | --- | --- | --- | --- | --- | --- | --- | --- | --- | --- |
| **Variable** | **Age** | |  | **Sex** | |  | **BMI** | |  | **PSQI** | |  | **AHI** | |  | **Group** | |  | **Time** | |  | **Group*Time** | |
|  | ***F (DF)*** | ***p*** |  | ***F (DF)*** | ***p*** |  | ***F (DF)*** | ***p*** |  | ***F (DF)*** | ***p*** |  | ***F (DF)*** | ***p*** |  | ***F (DF)*** | ***p*** |  | ***F (DF)*** | ***p*** |  | ***F (DF)*** | ***p*** |
| P-tau217 | 0.23 (1,12.9) | 0.640 |  | 2.01 (1,12.9) | 0.180 |  | 0.23 (1,12.9) | 0.639 |  | 0.77 (1,13) | 0.396 |  | 0.13 (1,12.9) | 0.722 |  | 6.06 (2,13) | **0.014** |  | 4.28 (8,21) | **0.0001** |  | 1.28 (16,121) | 0.219 |
| Aβ40 | 1.77 (1,12.9) | 0.206 |  | 0.04 (1,12.9) | 0.841 |  | 1.12 (1,12.9) | 0.310 |  | 0.03 (1,12.9) | 0.856 |  | 1.94 (1,12.9) | 0.187 |  | 0.1 (2,12.9) | 0.903 |  | 4.8 (8,121) | **<.0001** |  | 0.92 (16,121) | 0.553 |
| Aβ42 | 1.48 (1,12.9) | 0.246 |  | 0.16 (1,12.9) | 0.694 |  | 2.02 (1,12.9) | 0.179 |  | 0.99 (1,12.9) | 0.339 |  | 1.13 (1,12.9) | 0.308 |  | 0.51 (2,12.9) | 0.614 |  | 6.41 (8,121) | **<.0001** |  | 0.66 (16,121) | 0.830 |
| Aβ42/Aβ40 | 0.22 (1,13) | 0.644 |  | 0.25 (1,13) | 0.626 |  | 0.14 (1,13) | 0.718 |  | 1.38 (1,13) | 0.261 |  | 0.64 (1,13) | 0.438 |  | 0.79 (2,13) | 0.473 |  | 3.01 (8,21) | **0.004** |  | 0.99 (16,121) | 0.470 |
| GFAP | 5.45 (1,12.9) | **0.036** |  | 0.95 (1,12.9) | 0.349 |  | 0.04 (1,12.9) | 0.841 |  | 0.19 (1,12.9) | 0.669 |  | 0.48 (1,12.9) | 0.499 |  | 1.04 (2,12.9) | 0.383 |  | 1.88 (8,21) | 0.069 |  | 1.25 (16,121) | 0.242 |
| NfL | 3.94 (1,13) | 0.069 |  | 4.3 (1,13) | 0.059 |  | 0.18 (1,13) | 0.677 |  | 0.31 (1,13) | 0.586 |  | 0.61 (1,13) | 0.449 |  | 0.31 (2,13) | 0.742 |  | 2.05 (8,21) | **0.046** |  | 0.91 (16,121) | 0.557 |

**Supplementary Table 3. Mixed model results including age, sex, BMI, PSQI, and AHI in the model for evening and morning time points.**

|  |  | **Main effect** | | | | | | | | | | | | | | | | | | | | | | |
| --- | --- | --- | --- | --- | --- | --- | --- | --- | --- | --- | --- | --- | --- | --- | --- | --- | --- | --- | --- | --- | --- | --- | --- | --- |
| **Variable** | **N** | **Age** | |  | **Sex** | |  | **BMI** | |  | **PSQI** | |  | **AHI** | |  | **Group** | |  | **Time** | |  | **Group*Time** | |
|  |  | ***F (DF)*** | ***p*** |  | ***F (DF)*** | ***p*** |  | ***F (DF)*** | ***p*** |  | ***F (DF)*** | ***p*** |  | ***F (DF)*** | ***p*** |  | ***F (DF)*** | ***p*** |  | ***F (DF)*** | ***p*** |  | ***F (DF)*** | ***p*** |
| P-tau217 | 21 | 0.22 (1,12.9) | 0.647 |  | 2.38 (1,13) | 0.147 |  | 0.1 (1,12.9) | 0.757 |  | 0.76 (1,12.9) | 0.399 |  | 0.05 (1,12.9) | 0.819 |  | 5.23 (2,12.9) | **0.022** |  | 7.84 (1,17) | **0.0123** |  | 3.7 (2,17) | **0.046** |
| Aβ40 | 38 | 0.9 (1,29) | 0.350 |  | 0.11 (1,29.3) | 0.744 |  | 0.75 (1,29) | 0.393 |  | 0.02 (1,29) | 0.883 |  | 0.07 (1,29) | 0.795 |  | 0.66 (2,29.2) | 0.523 |  | 3.91 (1,32.7) | 0.0566 |  | 0.43 (2,32.7) | 0.657 |
| Aβ42 | 38 | 0.06 (1,29.4) | 0.804 |  | 0.04 (1,29.6) | 0.843 |  | 1.96 (1,29.4) | 0.172 |  | 0.26 (1,29.4) | 0.614 |  | 0 (1,29.4) | 0.963 |  | 0.47 (2,29.6) | 0.632 |  | 1.87 (1,32.8) | 0.1806 |  | 0.54 (2,32.8) | 0.589 |
| Aβ42/Aβ40 | 38 | 0.56 (1,29.8) | 0.459 |  | 0.04 (1,30) | 0.836 |  | 0.67 (1,29.8) | 0.420 |  | 0.88 (1,29.8) | 0.356 |  | 0.06 (1,29.8) | 0.807 |  | 0.07 (2,29.9) | 0.937 |  | 1.4 (1,33.1) | 0.245 |  | 0.17 (2,33.1) | 0.843 |
| GFAP | 38 | 8.73 (1,29.4) | **0.006** |  | 0 (1,29.4) | 0.957 |  | 0.01 (1,29.4) | 0.921 |  | 0.08 (1,29.4) | 0.784 |  | 0.03 (1,29.4) | 0.862 |  | 0.62 (1,29.4) | 0.546 |  | 2.78 (1,29.4) | 0.105 |  | 2.75 (1,29.4) | 0.079 |
| NfL | 38 | 11.44 (1,29.9) | 0.002 |  | 1.34 (1,30) | 0.256 |  | 0.43 (1,29.9) | 0.516 |  | 0.16 (1,29.9) | 0.694 |  | 0.07 (1,30) | 0.800 |  | 0.71 (2,30) | 0.500 |  | 0.28 (1,33.3) | 0.599 |  | 0.71 (2,33.3) | 0.500 |
